# Investigations into *Salmonella* Contamination in Feed Mills Producing Rations for the Broiler Industry in Great Britain

**DOI:** 10.3390/vetsci9070307

**Published:** 2022-06-21

**Authors:** Rebecca Gosling, Claire Oastler, Christopher Nichols, George Jackson, Andrew D. Wales, Robert H. Davies

**Affiliations:** 1 Department of Bacteriology, Animal and Plant Health Agency (APHA–Weybridge), Woodham Lane, New Haw, Addlestone KT15 3NB, UK; becky.gosling@apha.gov.uk (R.G.); chrisnichols@woodlandtrust.org.uk (C.N.); george.jackson@apha.gov.uk (G.J.); rob.davies@apha.gov.uk (R.H.D.); 2 Woodland Trust, Kempton Way, Grantham NG31 6LL, UK; 3 Department of Pathology and Infectious Diseases, School of Veterinary Medicine, University of Surrey, Guildford GU2 7AL, UK; a.wales@surrey.ac.uk

**Keywords:** poultry, broiler, feed, *Salmonella*, feed mill

## Abstract

Feed-associated *Salmonella* serovars continue to be reported in poultry flocks. A study was conducted to investigate *Salmonella* contamination in major commercial feed mills that produce rations for broiler chickens within Great Britain. Dust and large moist gauze swab samples (12,791) were collected from 22 feed mills on 31 visits. *Salmonella* was isolated from 20 mills, with 15 mills (75%) having fewer than 5% *Salmonella*-positive samples. Fifty-one *Salmonella* serovars were isolated, with a large proportion of isolates being *Salmonella* (*S*.) Kedougou (29.4%) or *S.* 13,23:i:- (21.4%). European Union-regulated *Salmonella* serovars (Enteritidis, Infantis, Typhimurium and its monophasic variants) were isolated from 12 mills, mostly from non-processing areas, accounting for 40 isolates (4.4% of all *Salmonella*-positive samples). Fifteen *Salmonella* serovars were only isolated once. In terms of individual sampling locations within the mill, the waste handling locations were significantly more likely to be *Salmonella*-positive than some other mill locations. When sampling locations were grouped, samples collected from finished product areas were significantly less likely to be *Salmonella*-positive for *Salmonella* than some other mill areas. In conclusion, this study found that most mills producing broiler rations showed low-level *Salmonella* contamination.

## 1. Introduction

The presence of *Salmonella* in animal feed is identified as a public health issue, with a risk of the organism being passed from the feed to food-producing animals and into the human food chain [1,2]. Monitoring for *Salmonella* contamination of compound animal feed production in the United Kingdom (UK) is carried out according to industry Codes of Practice [3]. Isolation of *Salmonella* from any feed sample should be reported to the competent authority.

Detecting *Salmonella* in feed ingredients and finished products can be problematic as compared with the large volume of feed, only small sample sizes are used and there is an uneven distribution of *Salmonella* within a consignment of feed. Even so, *Salmonella*-positive samples are reported regularly from feed ingredients and compound feed, and research studies have isolated *Salmonella* from samples collected from the feed production process and environment [4,5,6,7,8,9,10,11,12].

In 2018 in Great Britain (GB), there was an increase in *Salmonella* isolated from compound poultry feed compared to previous years, with *Salmonella* *(S.)* 13,23:i:- accounting for the largest proportion of isolates [13]. *Salmonella* serovars Kedougou and Senftenberg were second and third most common. *S.* 13,23:i:- is a monophasic variant of *S.* Idikan that increased over recent years and is frequently isolated from broiler production [14]. There were also three isolations of the regulated serovar *S.* Infantis. *Salmonella* was also isolated from individual feedstuff ingredients, with regulated serovars isolated from biscuit meal, minerals, wheat and soya (*S.* Infantis); malt and sunflower (*S.* Enteritidis) and palm kernel, soya and sunflower (*S.* Typhimurium). *S.* Enteritidis and *S.* Typhimurium were also isolated from feed mill environmental samples [13].

In GB, the most common *Salmonella* serovars reported from compound poultry feed were also isolated from poultry, specifically in the broiler sector. This link between the presence of *Salmonella* in feed and on-farm production is reported more frequently in poultry than in other livestock sectors, mainly because of the monitoring programmes in place which detect subclinical carriage of *Salmonella* [13]. For red meat species, only isolates associated with clinical disease are identified.

Contamination of animal feed with *Salmonella* can occur at many stages of its production. During growth of cereal crops, the use of contaminated irrigation water or manure, or the use of contaminated equipment can introduce the organism. Wildlife and pest species such as rodents can also contaminate feed during harvest or during storage and transportation [15,16,17,18]. Cereal-producing farms often also raise livestock, which can increase the risk of cross-contamination.

In feed mills there are additional contamination risks associated with the milling process. Some mills harbour resident *Salmonella* serovars which persist for many years. There may be reservoirs of such strains in specific pieces of milling equipment, but these organisms can disseminate throughout the mill by means of dust from contaminated equipment [9,10,12].

Risks also arise during the milling process as a result of using different raw ingredients from a range of national and international sources, combining *Salmonella*-contaminated and *Salmonella*-free ingredients during the mixing and subsequent production stages. Depending on the feed ration being produced, the milling process usually includes a heat treatment step which forms a critical control point for the control of *Salmonella* [12,19,20,21]. Failure to eliminate *Salmonella* from feed during this step allows contamination of subsequent cooling systems. These coolers can provide a favourable warm, moist environment for the multiplication of *Salmonella*, and thereby increase the risk of contamination of subsequent processing equipment and of compound feed.

The aim of the present study was to examine for *Salmonella* contamination of the environment and materials in feed mills producing rations for broiler chickens. Where a greater than 5% sample prevalence was detected, the distribution of *Salmonella* within the mill was assessed.

## 2. Materials and Methods

### 2.1. Premises

Twenty four feed mills producing broiler rations in GB were invited to take part in the study. These were selected to include major nationwide milling companies, integrated broiler company mills and independent mills. Twenty two of these (F1–F22) participated, representing the majority of British mills producing broiler rations. The remaining two mills were not included because of project time and resource constraints. Visits took place between September 2016 and November 2019. Fifteen mills received one visit, whilst seven mills were selected for longitudinal sampling. The number of repeat visits was determined by individual circumstances depending on the history of the mill in terms of *Salmonella* isolations from in-house monitoring or at previous visits carried out during this study. Additional visits were carried out where there were significant changes to the mill, such as changes in mill management, replacement of milling equipment, improvements to insulation or air filtering for coolers, or changes in feed treatments away from formaldehyde-based products. A total of two visits were made to mills F1, F2, F3, F4, F6 and F7, and four visits were made to mill F5. The interval between the repeat visits ranged from 1 month to 39 months.

### 2.2. Sampling

Sampling aimed to detect the presence of *Salmonella* in the feed mill environment with at least 95% confidence. The sampling size calculation (1-stage freedom analysis, https://epitools.ausvet.com.au/freedomss, (accessed on 2 May 2016) assumed an infinite population size, a 1% *Salmonella* prevalence in the feed mill environment, and the method described in the present study for the detection of *Salmonella* from feed and environmental samples being an imperfect test with a 100% test specificity and variable test sensitivity of 80–90%. This gave a sample size of 332–373 which was rounded up to 400 samples to be taken, where possible, at each mill visit. The sampling locations in the feed mill were categorised thus: intake pit, ingredient storage, ingredient handling, sieve, grinder, weigher/mixer, dust aspiration/cyclone, conditioner/press, cooler, crumbler, fat coater, finished product handling, finished product storage, outloading, and waste handling. Less localised sampling locations were: general interior environment, outside, and lorry wash/vehicles. From each mill 100 dust samples of 100 g were collected using gloved hands into new plastic jars (Medfor, Hampshire, UK). A new clean disposable glove was used for each sample. Dust samples comprised dusty material that emanated from specific equipment. The dust samples were collected throughout the mills from all sampling locations except for the lorry wash/vehicles location as no dust was present in this location. Each of the 100 dust samples was split into four 25 g samples for bacteriological analysis to give a total of 400 dust samples. However, there was some deviation due to mill-specific factors such as the size of the mill or the amount of dust present; the total number of dust samples collected from each mill ranged from 297 to 466. From the lorry wash/vehicles location, or other sampling locations where dust was sparse (or to compare dust samples and swab samples, data not shown), additional swab samples were also taken. From 28 of the 31 visits, between 10 and 142 swab samples were collected. Swab samples were collected using a large (900 cm^2^) sterile moist hand-held gauze swab (Robinson Healthcare LTD, Worksop, UK) that was immediately placed into 225 mL Buffered Peptone Water (BPW; Merck 10.07228.0500, Feltham, UK). The total number of samples (dust and swab) collected from each visit ranged from 317 to 496.

### 2.3. Bacteriological Analysis

Samples were processed using a method previously identified as being sensitive for the detection of *Salmonella* in feed and environmental samples [22]. Four samples (25 g) of the dust samples were each mixed with 225 mL BPW, resulting in four replicate samples. All samples in BPW were incubated at 37 ± 1 °C for 16 to 20 h, and then 0.1 mL of this pre-enriched sample was inoculated onto modified semi-solid Rappaport-Vassiliadis agar (MSRV; Mast DM440D, Bootle, UK) with 1 mg/ml added novobiocin (Sigma N1628, Sigma-Aldrich Company Ltd., Dorset, UK) and incubated at 41.5 ± 1 °C for 24 ± 3 h. Using a 1 µL loop, a subculture was taken from the edge of any opaque growth on the MSRV, inoculated onto Rambach agar (Merck 1.07500.0002, Feltham, UK) and incubated at 37 ± 1 °C for 24 ± 3 h. MSRV plates were returned to the incubator for a further 24 ± 3 h. Any MSRV plates on which growth enlarged between the 24 and 48 h incubations were sub-cultured again onto Rambach agar after 48 h incubation. All positive *Salmonella* isolates were confirmed by serotyping a single colony from each positive sample at the *Salmonella* reference laboratory at the Animal and Plant Health Agency (APHA) Weybridge, according to the White–Kauffmann–Le Minor scheme [22].

### 2.4. Statistical Analysis

For analysis, dust and swab samples are considered together and are referred to as ‘samples’. The proportion of *Salmonella*-positive samples was determined for each sampling location at each visit. Repeated chi-square tests were used to examine differences in the proportion of *Salmonella*-positive samples between each feed mill and the other feed mills. Analysis was performed in RStudio Version 1.3.1073 (RStudio Inc., Boston, MA, USA). To analyse the effect of individual sampling location and grouped sampling location on the prevalence of *Salmonella,* a generalised linear mixed-effects model with a binomial error structure was used. The random effects included were feed mill code, nested within visit number. Analysis was performed in Stata 15 (StataCorp LLC., College station, TX, USA).

## 3. Results

Two mills (F15 and F17) produced broiler rations only. The remaining mills also produced rations for other animals, particularly pigs and ruminants. One mill was independent and the rest formed part of different integrated broiler or feed companies, with eight different companies represented. Fifteen mills received one visit, whilst seven received repeat visits (Table 1).

### 3.1. Proportion of Salmonella-Positive Samples

The proportion of samples positive for *Salmonella* across the mills ranged from zero to 36.2% (Table 1). Comparing the proportion of *Salmonella*-positive samples between different mills showed a significant difference (chi-squared, *p* < 0.05) in the proportion of *Salmonella*-positive samples recovered between all mills. Fifteen mills yielded less than 5% *Salmonella*-positive samples on all sampling occasions, with two of these (visited once each) showing zero prevalence.

**Figure 1:**
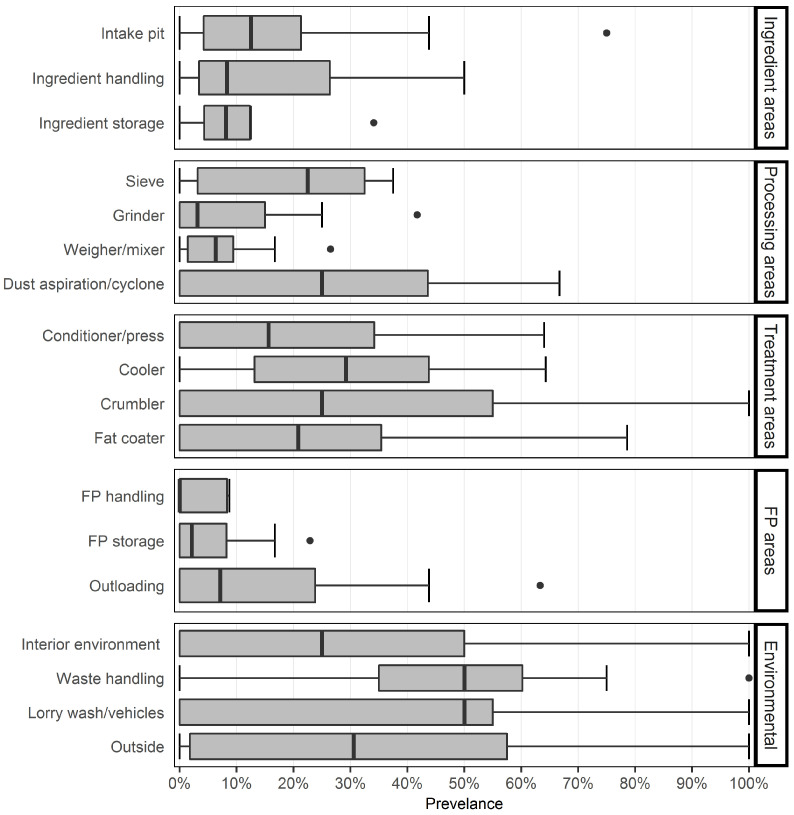
Boxplot of prevalence values of *Salmonella*-positive samples from feed mills, grouped by sampling location. Each box shows the median and interquartile range (IQR), and whiskers extend to the show the 2.5th percentile and the 97.5th percentile of the data. Outliers, data beyond the end of the whiskers, are plotted individually. Data are shown for feed mill visits where *Salmonella* was isolated from five percent or more of samples.

Further analysis was confined to visits where *Salmonella* was isolated from 5% or more of samples overall. Within this subset, the waste handling location showed the highest proportion of *Salmonella*-positive samples (49.6%) followed by the lorry wash/vehicles location (41.2%). Whilst samples from the finished product handling and the finished product storage locations showed the lowest proportion of positive samples, with values in both locations rarely exceeding 10% (Table 2 and Figure 1).

Comparisons were made of the prevalence of *Salmonella*-positive samples between all sampling locations individually, and also between groups of related sampling locations. These larger groups comprised ingredient areas (intake pit, ingredient handling, ingredient storage), processing areas (weigher/mixer, sieve, grinders, dust aspiration/cyclone), treatment areas (crumbler, conditioner/press, cooler, fat coater), finished product areas (finished product handling, finished product storage, outloading) and environmental areas (waste handling, interior environment, outside, lorry wash/vehicles). When each sampling location was considered separately, after the random effects of mill and sampling visit were taken into account, there was a significant association (glmm, *p* < 0.05) between some of the sampling locations and *Salmonella* prevalence. The outloading sampling location was used as the reference as the greatest number of samples were collected from this location. Samples taken from waste handling (odds ratio [OR] 7.03), lorry wash/vehicles (OR = 3.30), outside (OR = 2.67), crumbler (OR = 2.65), cooler (OR = 2.32), fat coater (OR = 1.76), and dust aspiration/cyclone (OR = 1.69) locations were significantly more likely (glmm, *p* < 0.05) to be *Salmonella*-positive than the outloading location. Samples taken from ingredient storage (OR = 0.54), grinder (OR = 0.49), weigher/mixer (OR = 0.36), finished product handling (OR = 0.30), and finished product storage (OR = 0.23) locations where significantly less likely (*p* < 0.05) to be *Salmonella*-positive than the outloading location. There was no significant difference in the proportions of *Salmonella*-positive samples between the outloading location and the conditioner/press (*p* = 0.06), sieve (*p* = 0.16), interior environment (*p* = 0.88), intake pit (*p* = 0.42), and ingredient handling (*p* = 0.45) locations; see Supplementary Material—Table S1. There was a significant association (glmm, *p* < 0.05) between some of the grouped sampling locations and *Salmonella* prevalence. The finished product areas were used as the reference as the greatest number of samples were collected from this grouped sampling location. Samples taken from the finished products areas were significantly less likely (glmm, *p* < 0.05) to be positive for *Salmonella* than the environmental areas (OR = 5.14), treatment areas (OR = 3.40), and processing areas (OR = 1.32). There was no significant difference in the proportions of *Salmonella*-positive samples between the finished product areas and the ingredient areas (*p* = 0.07); see Supplementary Material—Table S2.

### 3.2. Salmonella Serovars Recovered

**Figure 2:**
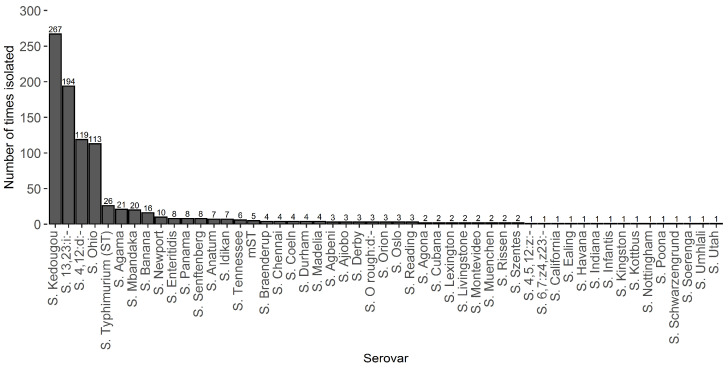
Number of times each *Salmonella* serovar was isolated across all 31 mill visits.

Fifty-one different *Salmonella* serovars were isolated across all of the mill visits (Figure 2). A large proportion of these were *S.* Kedougou and *S.* 13,23:i:-, originating from a small number of more highly contaminated mills. Fifteen serovars were isolated only once, including: *S.* 4,5,12:z:-, *S.* 6,7:z4,z23:-, *S.* California, *S.* Ealing, *S.* Havana, *S.* Indiana, *S.* Infantis, *S.* Kingston, *S.* Kottbus, *S.* Nottingham, *S.* Poona, *S.* Schwarzengrund, *S.* Soerenga, *S.* Umhlali and *S.* Utah.

**Figure 3:**
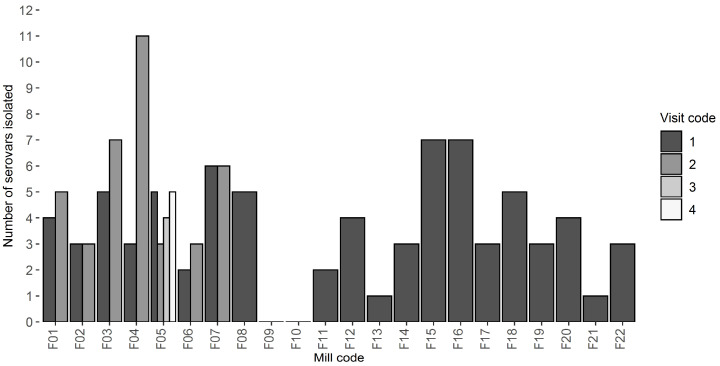
Number of different *Salmonella* serovars isolated from each mill at each of one to four visits.

The number of different serovars isolated from each *Salmonella*-positive mill at the first visit ranged from 1 to 11, with a mean value of four. Across all the mill visits, two mills yielded only one serovar whilst six mills had more than five serovars isolated. Of the mills receiving repeat visits, the number of serovars isolated did not differ greatly between visits (Figure 3). Among these mills, persistent serovars dominated the isolates, with the exception of F4 and F5, where the resident serovar was isolated infrequently and intermittently (Table 3). Different serotypes (*S.* Kedougou, *S.* 13,23:i:-, *S.* 4,12:d:- and *S.* Ohio) dominated across the different sampling locations, with S. Kedougou dominating in 12 of the 18 locations throughout the ingredient, processing, treatment, and environmental areas; see Supplementary Material—Table S3.

## 4. Discussion

The present study followed a similar sampling protocol to that used in previous investigations [23], focusing on the collection of dust in and around each piece of equipment, rather than analysing samples of feed. *Salmonella* is more frequently isolated from dust samples than from feed ingredients or compound feed in the same mills [11,21,24,25] and contaminated dust tends to settle locally around implicated equipment [26], therefore providing good localising information for contamination problems.

*Salmonella* was isolated from 20 of the 22 sampled feed mills. Of those mills where *Salmonella* was detected, 15 mills (75% of the total) showed a low level of contamination (fewer than 5% positive samples) at all visits, which is consistent with figures reported in a Brazilian feed mill study [24]. The highest prevalence of *Salmonella* in a feed mill in the present study (36.2%), is close to the highest prevalence values also reported from GB using a similar methodology in nine mills in 1997 (41.7%) and two mills in 2001 (37.4%) [9,23]. A more recent study of four British feed mills, again using a similar methodology, reported a maximum *Salmonella* prevalence value of 10.8% [12]. The median prevalence values for the two largest studies in GB, separated by two decades, are 11.7% [17] and 3.2% (present study). This suggests a possible general improvement of *Salmonella* control within British feed mills in that time, albeit that selection criteria differed between the two studies. However, the range of prevalence values in the current data indicates that *Salmonella* clearly remains an issue in some parts of the industry.

It is not surprising that a large number of serovars were isolated, due to the sources of the many ingredients used in feed formulations [12]. It is also to be expected that many serovars were not persistent, as survival of *Salmonella* in a feed mill is reported to be strain-dependent [27]. Persistent serovars isolated in the present study (*S.* 13,23:i:-, *S.* Ohio, *S.* Kedougou, and *S.* 4,12:d:-) correspond with commonly reported serovars from contemporary GB poultry feed surveillance data [13]. Furthermore, *S.* 13:23:i:- and *S.* Kedougou were the first and third most common serovars isolated in broiler flocks in 2018 [13], which is consistent with contaminated feed rations being a source of *Salmonella* contamination of broilers, as previously documented [28].

Some key *Salmonella* serovars of public health importance targeted by the National Control Programme (*S.* Enteritidis, *S.* Infantis, *S.* Typhimurium and its monophasic variant) were isolated in the present investigation. However, they accounted for fewer than 5% of samples overall and were mostly found in ingredient and non-production-environment samples, rather than production or finished product areas where only a few isolates were found. The serovars *S.* Enteritidis and *S.* Typhimurium do not persist in feed mill environments, but these serovars may intermittently be isolated from feed ingredients, as observed in the present study, or affect wild birds in the mill environment. There is no proven case of feed being a source of these particular serovars in broilers, but occasional cases are likely, particularly for laying hens for which feed is not heat treated [29].

In the present study the distribution of *Salmonella* throughout the feed mill was only assessed for mills with a *Salmonella* prevalence of 5% or greater, in order that findings reflected the situation in mills with substantial or persistent *Salmonella* contamination. Waste handling and lorry wash/vehicles were the individual sampling locations with the highest proportion of positive samples and were significantly more likely (glmm, *p* < 0.05) to be *Salmonella*-positive than some of the other sampling locations in the mill. It can be appreciated that neither area may receive the focused attention to hygiene, including cleaning down and dust and moisture control, which may be present in the core processing pathway. Furthermore, both areas will regularly be exposed to ingredients before their effective microbicidal heat treatment as well as visiting vehicles and personnel.

The finished product areas had the lowest proportion of *Salmonella*-positive samples and were significantly less likely (glmm, *p* < 0.05) to be *Salmonella*-positive than some of the other mill areas. This probably reflects a number of protective factors, including recent heat treatment and drying steps, a protected environment away from untreated ingredients, machinery and wildlife, as well as an environment where dust and moisture control is easier to accomplish than in unloading and processing stages. In contrast, the sampling locations with the highest proportion of *Salmonella* isolation included the pellet coolers; indeed, all visits yielding a prevalence of positive samples in excess of 10% showed substantial cooler contamination. Coolers commonly present intractable contamination due to several factors including moisture, warmth, high airflow and difficult access for cleaning.

Intake pits and augers were previously reported to be the feed mill areas with the highest proportion of *Salmonella*-positive samples [9,11]. However, these studies collected samples from mills producing feed for several species. Data from poultry feed mills only, within the report by Davies and Wray [9], showed coolers to be heavily contaminated, with 61% of samples yielding *Salmonella*, followed by the intake pits and augers, with 47% positive samples. In poultry feed mills investigated by Davies and Wales [12] the ingredient areas showed most frequent contamination, with contamination of coolers being identified in certain mills only.

The repeated sampling visits in the present investigation (Table 3) were conducted in a variety of scenarios, but contamination of coolers was a frequent issue. Two of the mills had previously been investigated for persistent contamination by resident serovars. F1 had been affected by *S.* Binza and *S.* Ohio [23], whilst F4 was colonised by *S.* Kedougou [12]. The repeated sampling demonstrated that this contamination had cleared (F1) or dramatically reduced (F4), albeit not tested for statistical significance, following the addition of insulation and air filtration to pellet cooling systems. Mill F5 experienced a recurrence of cooler contamination by *S.* Ohio after maintenance work in the mill and the deterioration of cooler insulation, as well as stopping the use of formaldehyde-based feed additives. Mills F3 and F6 reduced their contamination following improved hygiene procedures, but still needed to apply insulation and filtration to coolers. Mills F2 and F7 experienced massive amplification, albeit not tested for statistical significance, of the resident strains present in their pellet cooling systems after major maintenance projects or dust clearing work in the mills.

The importance of the need for *Salmonella* control within pellet coolers is evident in the above cases; the key control factor appears to be not allowing the temperature at the entry point of the cooler to drop to *Salmonella*-multiplication levels. This can be achieved by better control of conditioning times and temperature dips and a high standard of insulation between the pellet press and the cooler discharge point, which also helps to minimise condensation. Filtration of the cooler air intakes also helps reduce the risk of airborne *Salmonella* ingress.

There is great concern in the feed industry regarding the potential detrimental effect of banning formaldehyde-containing feed additives [14,30]. Five mills in the present study were sampled before and after they stopped using such additives; three of these exhibited an increase in contamination when resampled. However, in all these mills other factors that may contribute to worsened contamination also applied and contamination in the other two mills improved between visits.

In conclusion, although the introduction of *Salmonella* into feed mills is most likely to be via contaminated feed entering the plant, the thermal processes in place to eliminate and prevent further contamination continue to face challenges. These include cooler contamination, dust control and external agents such as visiting vehicles or wild birds. Those mills with cooler contamination tend to act as multipliers of *Salmonella* contamination, resulting in more contamination of finished product than ingredients and greater likelihood of infecting broilers. There is wide variation in the degree of *Salmonella* contamination within GB broiler feed mills, which is substantially a consequence of the extent of cooler contamination by resident *Salmonella* strains. In view of repeated isolation of the same serovar from coolers and related areas of the mills, but not in the ingredient areas, it is hypothesised that cooler contamination can persist for years but molecular typing is needed to confirm this.

## Figures and Tables

**Table 1:** Proportion of *Salmonella*-positive samples from each visit to 22 mills producing broiler feed.

Feed Mill Code	Visit Number	Number of Samples	Proportion of *Salmonella*-Positive Samples
F1	1	393	1.3 (CI 0.5–3.0)
	2	496	1.4 (CI 0.7–2.9)
F2	1	386	2.1 (CI 1.0–4.0)
	2	420	**29.5 (CI 25.4–34.1)**
F3	1	381	**15.0 (CI 11.7–18.9)**
	2	400	**12.8 (CI 9.8–16.4)**
F4	1	486	1.2 (CI 0.6–2.7)
	2	445	3.4 (CI 2.0–5.5)
F5	1	407	**5.4 (CI 3.6–8.0)**
	2	416	**5.3 (CI 3.5–7.9)**
	3	412	2.2 (CI 1.2–4.1)
	4	435	4.8 (CI 3.2–7.3)
F6	1	420	**36.2 (CI 31.7–40.9)**
	2	352	**19.6 (CI 15.8–24.1)**
F7	1	416	**12.7 (CI 9.9–16.3)**
	2	454	**35.9 (CI 31.6–40.4)**
F8	1	380	1.3 (CI 0.6–3.0)
F9	1	404	0.0 (CI 0–0.9)
F10	1	410	0.0 (CI 0–0.9)
F11	1	428	0.7 (CI 0.2–2.0)
F12	1	420	1.2 (CI 0.5–2.8)
F13	1	420	0.2 (CI 0.0–1.3)
F14	1	372	3.5 (CI 2.0–5.9)
F15	1	405	**6.9 (CI 4.8–9.8)**
F16	1	440	3.4 (CI 2.1–5.6)
F17	1	317	3.2 (CI 1.7–5.7)
F18	1	424	1.7 (CI 0.8–3.4)
F19	1	408	1.0 (CI 0.4–2.5)
F20	1	390	1.3 (CI 0.6 – 3.0)
F21	1	439	0.2 (CI 0.0–1.3)
F22	1	415	**6.3 (CI 4.3–9.0)**

Numbers in bold highlight where the percentage of *Salmonella*-positive samples was 5% or more. CI = 95% confidence interval for the proportion of *Salmonella*-positive samples.

**Table 2:** Prevalence of *Salmonella*-positive samples by location, for feed mill visits where the organism was isolated from five percent or more of samples.

Mill Code (F)/Visit Code (v)	Intake Pit	Ingredient Handling	Ingredient Storage	Sieve	Grinder	Dust Aspiration/Cyclone	Weigher/Mixer	Conditioner/Press	Cooler	Crumbler	Fat Coater	Finished Product Handling	Finished Product Storage	Outloading	Interior Environment	Waste Handling	Lorry Wash/Vehicles	Outside
F2/v2	24.4 (45)	0.0 (4)	11.8 (34)	37.5 (40)	3.1 (32)	25.0 (8)	6.3 (32)	33.3 (36)	50.0 (28)	25.0 (4)	78.6 (28)	0.0 (4)	2.8 (36)	27.6 (58)	100 (4)	75.0 (20)	60.0 (5)	50.0 (2)
F3/v1	11.5 (52)	8.3 (36)	0.0 (24)	30.0 (20)	nd	50.0 (8)	2.8 (36)	15.6 (32)	29.2 (24)	nd	25.0 (12)	8.7 (46)	16.7 (18)	17.9 (56)	50.0 (6)	50.0 (2)	nd	11.1 (9)
F3/v2	3.6 (56)	nd	9.4 (64)	25.0 (24)	0.0 (15)	66.7 (12)	16.7 (36)	28.1 (32)	29.2 (24)	nd	20.7 (29)	nd	0.0 (40)	0.0 (56)	25.0 (4)	0.0 (8)	nd	nd
F5/v1	43.8 (16)	8.3 (84)	3.8 (52)	0.0 (8)	10.0 (20)	0.0 (24)	0.0 (20)	0.0 (16)	0.0 (36)	0.0 (4)	0.0 (8)	0.0 (8)	0.0 (60)	0.0 (28)	0.0 (12)	40.0 (10)	0.0 (1)	nd
F5/v2	0.0 (16)	2.4 (84)	1.9 (52)	0.0 (8)	0.0 (20)	12.5 (24)	6.3 (16)	0.0 (16)	19.4 (36)	25.0 (4)	37.5 (8)	0.0 (8)	6.7 (60)	0.0 (32)	0.0 (16)	0.0 (8)	0.0 (3)	0.0 (5)
F6/v1	4.2 (24)	29.2 (48)	34.1 (44)	12.5 (8)	25.0 (12)	25.0 (8)	6.3 (32)	50.0 (24)	60.0 (20)	75.0 (24)	20.8 (24)	8.3 (12)	22.9 (48)	63.3 (60)	50.0 (10)	50.0 (4)	50.0 (2)	56.3 (16)
F6/v2	4.2 (24)	17.9 (28)	6.8 (44)	33.3 (12)	15.0 (20)	0.0 (8)	12.5 (32)	35.0 (20)	29.2 (24)	55.0 (20)	33.3 (24)	0.0 (8)	2.1 (48)	20.0 (20)	75.0 (4)	33.3 (12)	nd	100 (4)
F7/v1	18.2 (44)	50.0 (8)	12.5 (64)	20.0 (20)	0.0 (12)	0.0 (16)	0.0 (44)	0.0 (16)	37.5 (32)	0.0 (12)	0.0 (16)	nd	0.0 (48)	7.1 (56)	0.0 (8)	nd	100 (2)	61.1 (18)
F7/v2	12.5 (32)	50.0 (8)	12.5 (32)	35.0 (20)	41.7 (24)	45.5 (22)	26.5 (34)	64.0 (25)	64.3 (56)	100 (6)	50.0 (16)	nd	9.7 (72)	43.8 (73)	9.5 (21)	63.6 (11)	50.0 (2)	nd
F15/v1	75.0 (16)	6.3 (48)	5.8 (69)	nd	0.0 (20)	0.0 (8)	0.0 (20)	0.0 (16)	0.0 (36)	0.0 (20)	0.0 (8)	8.3 (12)	0.0 (96)	0.0 (20)	nd	100 (8)	nd	0.0 (8)
F22/v1	12.5 (40)	0.0 (16)	nd	0.0 (20)	nd	41.7 (12)	6.3 (16)	0.0 (18)	6.9 (29)	0.0 (8)	0.0 (16)	0.0 (16)	0.0 (16)	0.0 (140)	nd	50.0 (24)	0.0 (2)	2.4 (42)

Values are percent positive samples. Total number of samples are in brackets. ‘nd’ indicates no data.

**Table 3:** Occurrence of *Salmonella* serovars at repeat visits.

Mill	Persistent Serovar	Visit	Number of *Salmonella*-Positive Samples	
			Persistent Serovar	Other Serovars
F1	None	1	-	5
		2	-	7
F2	*S.* 4,12:d:-	1	4	4
		2	115	9
F3	*S.* Ohio	1	38	19
		2	35	16
F4	*S.* Kedougou	1	3	3
		2	0	15
F5	*S.* Ohio	1	0	22
		2	19	3
		3	6	3
		4	15	6
F6	*S.* Kedougou	1	145	7
		2	66	3
F7	*S.* 13,23:i:-	1	35	11
		2	152	18

## Data Availability

Some additional data from this study are available in Supplementary Material—Tables S1–S3.

## Supplementary Materials

The following supporting information can be downloaded at: https://www.mdpi.com/article/10.3390/vetsci9070307/s1, Table S1: Results of the binomial generalized linear mixed model for individual sampling locations; Table S2: Results of the binomial generalized linear mixed model for grouped sampling locations; Table S3: Dominant *Salmonella* serovars by sampling location.
