# Supplementary material for: Investigations into Salmonella Contamination in Feed Mills Producing Rations for the Broiler Industry in Great Britain
Source: Vet Sci. 2022 Jun 21;9(7):307. doi: 10.3390/vetsci9070307 (PMC9323917; doi:10.3390/vetsci9070307)
Supplement: Supplementary file 1 [file vetsci-09-00307-s001.zip › vetsci-1716416-supplementary.pdf]

Supplementary material - Table S1. Results of the binomial generalized linear mixed model for individual sampling locations

Mixed-effects logistic regression, Number of observations = 4,486

Group variable	No. of observations per group	Minimum	Average	Maximum
Mill	7	405	640.9	870
Visit	11	352	407.8	454

Integration method: mvaghermite, Integration points = 7

Wald chi2(17) = 276.74, Log likelihood = -1707.425, Prob > chi2 = 0.0000

Sampling location	Odds ratio	Std.	Error	z value	P>|z|	95% confidence interval
Conditioner/press	1.432083	0.360726	0.194278	1.86	0.063	-0.0200529, 0.7415043
Cooler	2.318128	0.837753	0.173319	4.83	0	0.4980531, 1.177453
Crumbler	2.653209	0.969804	0.257312	3.77	0	0.4654821, 1.474127
Dust aspiration/cyclone	1.693047	0.522454	0.241855	2.16	0.031	0.048426, 0.9964812
Fat coater	1.759272	0.565456	0.206378	2.74	0.006	0.1609632, 0.969948
Finished product handling	0.298481	-1.21028	0.445981	-2.71	0.007	-2.084381, -0.3361683
Finished product storage	0.229088	-1.47335	0.23296	-6.32	0	-1.929937, -1.016752
Interior environment	1.043562	0.046158	0.30706	0.15	0.881	-0.5556674, 0.6479836
Grinder	0.487771	-0.71629	0.277122	-2.58	0.01	-1.259441, -0.1731417
Ingredient handling	0.85391	-0.16344	0.214051	-0.76	0.445	-0.5829734, 0.2560919
Ingredient storage	0.536489	-0.62462	0.197355	-3.16	0.002	-1.011431, -0.2378129
Intake pit	0.858963	-0.15156	0.189559	-0.8	0.424	-0.5230936, 0.2199652
Lorry wash/vehicles	3.298903	1.192485	0.562774	2.12	0.034	0.0894691, 2.295501
Outside	2.671286	0.96621	0.276274	3.5	0	0.4247228, 1.507698
Sieve	1.353075	0.304434	0.21793	1.4	0.162	-0.1226999, 0.7315679
Waste handling	7.034172	1.939168	0.249633	7.77	0	1.449897, 2.428438
Weigher/mixer	0.358094	-1.02627	0.239107	-4.29	0	-1.494913, -0.5576293
Outloading (reference)	-	-1.89221	0.342583	-5.52	0	-2.563661, -1.22076

--------------+--

Mill |
var(_cons) | .458338, .445635 .0681672, 3.081741

--------------+--

Mill>Visit |
var(_cons) | .375851, .2817317 .0864906, 1.633286

LR test vs. logistic model: chi2(2) = 383.10, Prob > chi2 = 0.0000
Note: LR test is conservative and provided only for reference.

Akaike's information criterion and Bayesian information criterion

--------------+--

Model	Obs ll(null	ll(model)	df	AIC	BIC
.	4,486	.-1774.573	20	3454.85	3583.02

Note: N=Obs used in calculating BIC; see [R] BIC note.

Supplementary material - Table S2. Results of the binomial generalized linear mixed model for grouped sampling locations

Mixed-effects logistic regression, Number of observations = 4,486

Group variable	No. of observations per group	Minimum	Average	Maximum
Mill	7	405	640.9	870
Visit	11	352	407.8	454

Integration method: mvaghermite, Integration pts. = 7

Wald chi2(4) = 185.26, Log likelihood = -1774.5729, Prob > chi2 = 0.0000

Grouped sampling location	Odds ratio	Std.	Error	z value	P>|z|	95% confidence interval
Environmental areas	5.144869	1.631392	0.163753	9.96	0	1.310443, 1.952341
Ingredient areas	1.271631	0.238616	0.132329	1.8	0.071	-0.0207454, 0.4979765
Processing areas	1.323659	0.281817	0.139336	2.02	0.043	0.008724, 0.5549091
Treatment areas	3.403145	1.224073	0.123584	9.9	0	0.9818519, 1.466293
Finished product areas (reference)		-2.42323	0.317743	-7.63	0	-3.045994, -1.800463

-------------+--

Mill |

var(_cons) | .390615,.3957866 .053614, 2.845902

-------------+--

Mill>Visit |

var(_cons) | .3565619,.2648157 .0831676, 1.528677

LR test vs. logistic model: chi2(2) = 364.74, Prob > chi2 = 0.0000

Note: LR test is conservative and provided only for reference.

Akaike's information criterion and Bayesian information criterion

----

Model	Obs ll(null	ll(model)	df	AIC	BIC
.	4,486	.-1774.573	7	3563.146	3608.007

Note: N=Obs used in calculating BIC; see [R] BIC note.

Supplementary material - Table S3. Dominant *Salmonella* serovars by sampling location

Grouped sampling location	Sampling location	Dominant serovar	Number of *Salmonella*-positive samples	
			Dominant serovar	Other serovars
Ingredient areas	Intake pit	*S.* 13,23:i:-	12	72
	Ingredient handling	*S.* Kedougou	21	40
	Ingredient storage	*S.* Kedougou	19	54
Processing areas	Sieve	*S.* 4,12:d:-	15	29
	Grinder	*S.* 13,23:i:-	10	9
	Dust aspiration/cyclone	*S.* Kedougou	14	27
	Weigher/mixer	*S.* Kedougou	10	21
Treatment areas	Conditioner/press	*S.* Kedougou	21	44
	Cooler	*S.* Kedougou	46	72
	Crumbler	*S.* Kedougou	28	11
	Fat coater	*S.* 4,12:d:-	22	37
Finished product areas	Finished product handling	*S.* Ohio	4	2
	Finished product storage	*S.* Kedougou	13	17
	Outloading	*S.* Kedougou	46	64
Environmental	Interior environment	*S.* Kedougou	8	10
	Waste handling	*S.* Kedougou	18	45
	Lorry wash/vehicles	*S.* 4,12:d:-	3	3
		S. 13,23:i:-	3	
	Outside	*S.* Kedougou	8	29
